# RNA-Seq using bulked recombinant inbred line populations uncovers the importance of brassinosteroid for seed longevity after priming treatments

**DOI:** 10.1038/s41598-017-08116-5

**Published:** 2017-08-14

**Authors:** Naoto Sano, June-Sik Kim, Yoshihiko Onda, Takahito Nomura, Keiichi Mochida, Masanori Okamoto, Mitsunori Seo

**Affiliations:** 1 RIKEN Center for Sustainable Resource Science, 1-7-22 Suehiro-cho, Tsurumi-ku, Yokohama, Kanagawa 230-0045 Japan; 2 Arid Land Research Center, Tottori University, 1390 Hamasaka, Tottori, 680-0001 Japan; 3 Center for Bioscience Research and Education, Utsunomiya University, 350, Mine-machi, Utsunomiya, 321-8505 Japan; 4 Kihara Institute for Biological Research, Yokohama City University, 641-12 Maioka-cho, Totsuka-ku, Yokohama, Kanagawa 244-0813 Japan; 5 Institute of Plant Science and Resources, Okayama University, 2-20-1 Chuo, Kurashiki, Okayama, 710-0046 Japan; 6 RIKEN Center for Sustainable Resource Science, 3-1-1 Koyadai, Tsukuba-shi, Ibaraki 305-0074, Japan (JS. K.); Center for Bioscience Research and Education, Utsunomiya University, 350, Mine-machi, Utsunomiya, 321-8505 (M.O.) Japan

## Abstract

Seed priming is a commercially used technique for improving seed performance including germination. However, the treatment sometimes reduces seed longevity as a side effect, limiting the storable period or longevity of the seeds. To overcome this problem, molecular mechanisms involved in the loss of seed longevity during priming were analyzed using natural variations of *Arabidopsis thaliana*. We found that the Est-1 accession retained longevity for longer after priming compared to the reference accession Col-0. QTL analysis using 279 recombinant inbred lines (RILs) derived from the Est-1 × Col-0 detected three QTL regions associated with the loss of seed longevity during priming. Bulked transcriptome analysis (RNA-Seq with bulked RIL populations) revealed that genes related to brassinosteroid (BR) biosynthesis/signaling and cell wall modification were highly expressed in primed seeds with shorter longevity. After priming, BR-deficient mutants *cyp85a1/a2* and *det2* showed significantly longer longevity than the wild type (WT). Moreover, tetrazolium staining indicated that mutant seed coats were less permeable after priming than those of WT. We suggest that the loss of seed longevity in primed seed is due to increased seed coat permeability, which is positively regulated, at least partly, via BR signaling.

## Introduction

Germination is the process during which a quiescent dry seed become a seedling. Germination commences with imbibition of dry seeds, followed by expansion of embryonic cells and rupture of the surrounding layers: endosperm and seed coat (testa). The completion of germination is often defined as radicle emergence from the surrounding layers, whereas dormancy represents a physiological status that enables viable seeds not to germinate even under favorable conditions. Although dormancy is an important trait especially for wild species for their survival in changing environments, extreme dormancy often causes poor germination or non-synchronous germination and the trait is detrimental for crop cultivation. Therefore, less dormant (or rapid germination) traits have been preferentially selected over time through breeding, especially for cereals. However, this can enable pre-harvest sprouting (viviparous germination or precocious germination) which reduces the quality of the products (seeds) not only as foods but also as individuals for the next generation. The precise regulation of these two opposing physiological processes, dormancy and germination, is thus desirable for efficient agricultural production.

Seed priming is a commercially used technique to improve seed performance including germinability, uniformity, vigor and stress tolerance. The treatment involves imbibition of seeds in water under controlled conditions to trigger the metabolic processes that are normally activated during the early phase of germination (pre-germinative metabolism) and subsequent drying of the seeds prior to full germination, or radicle emergence, so that seeds reenter a quiescent state^1, 2^. Several kinds of priming treatments such as hydropriming, osmopriming, solid matrix priming and biopriming have been developed^2^. Among them, osmopriming is a widespread pre-sowing procedure that involves treatments with osmotic solutions such as polyethylene glycol or inorganic salts of sodium, potassium and magnesium (most commonly NaCl, NaNO_3_, MnSO_4_, MgCl_2_, K_3_PO_4_ and KNO_3_) at low water potential facilitating controlled water uptake. In contrast, hydropriming is simple and the most traditional type of priming, with seeds soaked in water under optimal temperature conditions (usually in a range from 5 to 20 °C) for a set time period. Imbibition at high or low temperature before sowing promotes germination depending on plant species and this is known as thermopriming. Besides the benefits of priming to enhance seed germination, the treatments applied sometimes reduce seed longevity (total time span during which seeds remain viable) or storability^3^. Primed seeds with high performances in terms of germination are thought to have a more advanced physiological status in the germination process compared with seeds without priming, and are thus more susceptible to deterioration^1^. This loss of seed longevity is a disadvantage of priming with regard to commercial distribution.

The molecular aspects of seed longevity have been reviewed^4–6^. During dry storage of mature seeds, reduction of longevity is mainly caused by oxidation of cellular macromolecules such as nucleic acids, proteins and lipids. To circumvent the effects, seeds possess protective mechanisms such as the formation of a glassy cytoplasm to reduce cellular metabolic activities and the production of antioxidants, as well as repair systems that remove damage accumulated in DNA, RNA and proteins upon seed imbibition through enzymes such as DNA glycosylase and methionine sulfoxide reductase. In addition, the seed coat represents the interface through which the embryo interacts with the external environment and thus its composition and structure are critical factors for seed longevity. Several factors that determine seed longevity during dry storage have been identified so far, however, little is known about the molecular mechanisms involved in the loss of seed longevity during priming treatments.

*Arabidopsis* is not only a model plant but also a member of one of the largest families of flowering plants, the Brassicaceae, which includes several crop species such as mustard, radish, cauliflower, broccoli and cabbage. Natural variations (also referred to as ecotypes or accessions) of *Arabidopsis* have tremendous genetic and phenotypic diversity, and QTL mapping has been successfully employed to identify genes that are responsible for the variations in a range of physiological and developmental traits^7^. Among several mapping population that can be used for QTL analysis, recombinant inbred lines (RILs) offer unique advantages: homozygous lines that can be propagated without genetic changes^8^ allow us to conduct replicate experiments to evaluate their phenotypes.

In this study, we compared seed longevity of 230 *Arabidopsis* natural variations before and after priming and identified an accession, Est-1, which can retain a relatively high level of seed longevity after priming when compared with the reference accession, Col-0. Mapping with a RIL population derived from a cross between Col-0 and Est-1 detected three major QTLs associated with the loss of seed longevity during priming. Furthermore we performed transcriptome analysis with the RILs to predict genes that might be involved in the regulation of seed longevity. The relationship between seed coat permeability as modulated by the plant hormone brassinosteroid (BR) and the loss of seed longevity during priming will be discussed.

## Results

### Effects of priming on seed germination and longevity

**Figure 1:**
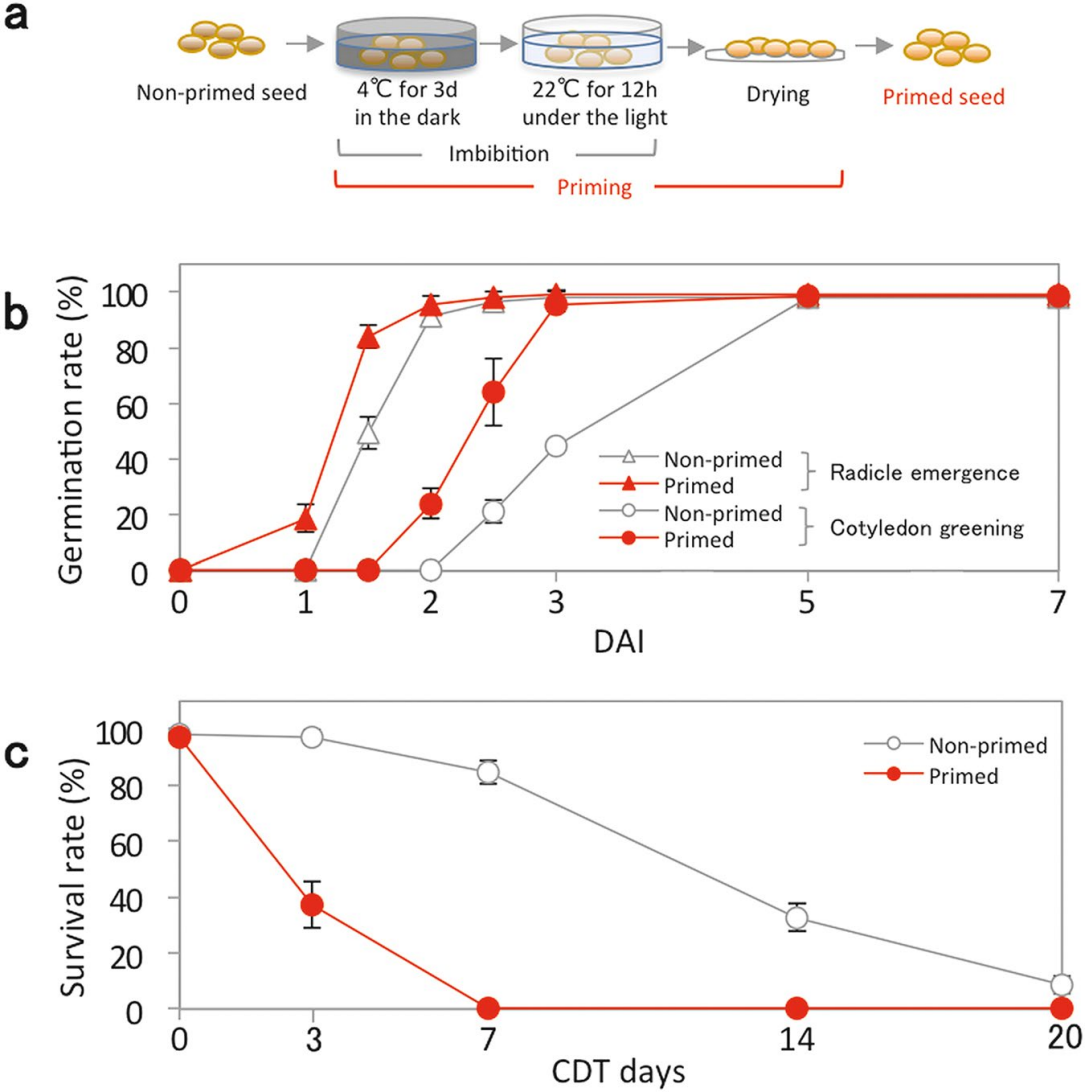
Effect of priming on germination speed and seed longevity. (**a**) A simplified scheme for the priming treatment used in this study. (**b**) Germination speed of primed and non-primed seeds without CDT determined as radicle emergence from the seed coat or cotyledon greening. (**c**) Survival rates of primed and non-primed seeds after 0, 3, 7, 14 and 20 days of CDT. Values are means ± SD of three replicates for (**b**) and (**c**).

We first set up an experimental system to determine the effects of priming on seed longevity using the *Arabidopsis* Col-0 reference accession (Fig. 1a). Although Col-0 is known as a weakly dormant accession, germination behavior (e.g. germination speed and final germination percentage) of the seeds varies depending on environmental conditions^9^. To eliminate the effects caused by variable dormancy status and to synchronize germination, mature dry seeds that had been after-ripened for two to four months were imbibed at 4 °C for 3 days in the dark. This treatment is generally called stratification, but can also be considered as a part of priming (thermopriming). Then the seeds were incubated at 22 °C in the light for 12 h to partially initiate germination events; this treatment is considered as hydropriming. Finally, seeds were dried to a quiescent physiological state. In this manuscript, we refer to the combined series of these treatments as priming. Seeds did not complete germination during these treatments as testa rupture or radicle emergence was not observed. Upon imbibition, radicle protrusion and subsequent establishment of seedlings with green cotyledons was much faster for primed seeds than non-primed seeds (Fig. 1b). Furthermore, the primed seeds were more resistant to high temperature in terms of radicle protrusion compared with non-primed seeds (Supplementary Fig. S1). These observations demonstrated that the priming treatment used in this study effectively enhanced germination performance. Hereafter, we have scored cotyledon greening in germination tests rather than radicle protrusion because this is a better representation of the effect of priming on seedling survival and establishment.

To evaluate the effects of priming on seed longevity, we employed a controlled deterioration treatment (CDT); the sensitivity or resistance of seeds to CDT can be used for rapid assessment and prediction of seed longevity during natural aging^10, 11^. Primed and non-primed seeds were subjected to CDT by incubating the seeds at 37 °C under 80% relative humidity for 0, 3, 7, 14 and 20 days. After CDT, the ability of seeds to establish healthy seedlings with green cotyledons upon imbibition was scored as the survival rate (Fig. 1c). Note that individuals that did not grow into healthy seedlings after radicle protrusion were not considered as survival. Survival rates of the non-primed seeds were not significantly impacted after 3 days of CDT, and more than 80% of non-primed seeds could still establish healthy seedlings after 7 days of CDT. In contrast, survival rates of primed seeds decreased rapidly to 40% within 3 days of CDT, and none of the primed seeds survived after 7 days of CDT. These data indicate that negative impacts of priming on seed longevity can be determined by this experimental system.

### Effects of dormancy and endogenous abscisic acid (ABA) levels on the longevity of primed seeds

**Figure 2:**
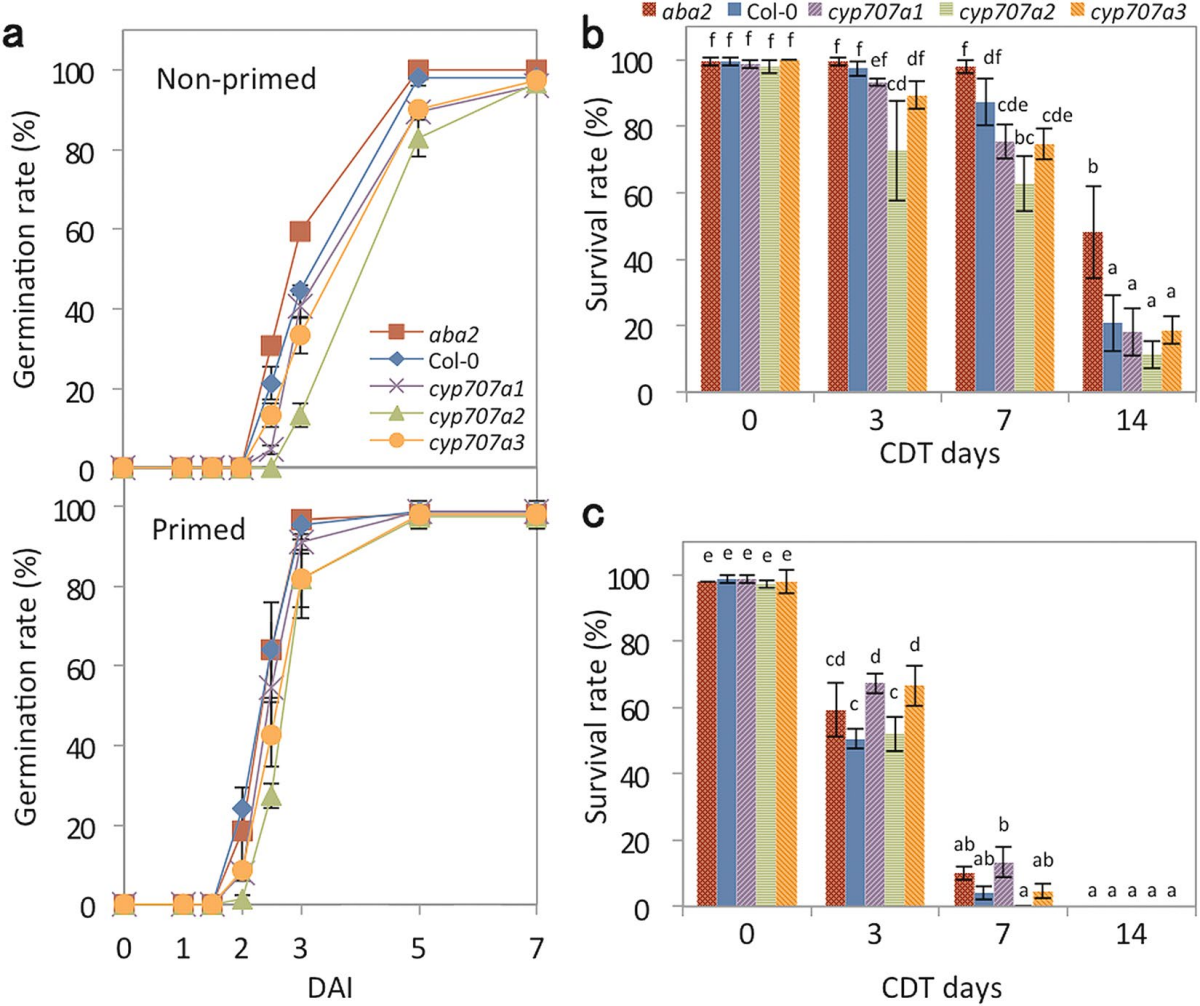
Effect of priming on germination and longevity of ABA-deficient and ABA over-accumulating mutants. (**a**) Germination speed of wild type Col-0 (Col-0), *aba2*, *cyp707a1*, *cyp707a2* and *cyp707a3* before (non-primed seeds; upper panel) and after (primed; lower panel) priming without CDT. (**b**) Survival rates of non-primed Col-0, *aba2*, *cyp707a1*, *cyp707a2* and *cyp707a3* seeds after 0, 3, 7 and 14 days of CDT. (**c**) Survival rates Col-0, *aba2*, *cyp707a1*, *cyp707a2* and *cyp707a3* seeds after priming and 0, 3, 7 and 14 days of CDT. Values presented are means ± SD of three replicates. Different letters in (**b**) and (**c**) indicate significant differences (P < 0.05, Tukey-Kramer tests).

An earlier study had shown that the *Arabidopsis* ABA deficient mutant *aba1-5* in the Columbia genetic background had reduced seed dormancy and longevity compared to the wild type (WT)^12^. Conversely, QTL analysis using six *Arabidopsis* RIL populations showed that natural variations for dormancy and longevity were negatively correlated^13^. The relationship between seed dormancy and seed longevity were investigated in our experimental conditions, by determining the germination speed and seed longevity of ABA deficient and ABA over-accumulating mutants in the Columbia background. The *aba2* mutant is defective in an ABA biosynthesis enzyme, whereas *cyp707a* mutants accumulate high levels of ABA compared with WT due to their defective ABA catabolism^14–16^. Although all genotypes tested germinated to around 100% within 7 days after imbibition (DAI), faster germination was still observed for non-primed *aba2* seeds compared to WT, indicating that *aba2* was less dormant than WT (Fig. 2a). In contrast, seeds of *cyp707a* mutants, especially *cyp707a2*, germinated more slowly than WT seeds. This is consistent with previous results that showed *cyp707a2* seeds are more dormant than those of *cyp707a1* and *cyp707a3* ^16^. We then examined longevity, or survival rates, of the seeds after CDT. We found that seedling survival for *aba2* was higher than WT after 14 days of CDT, whereas *cyp707a2* was more sensitive to CDT and exhibited lower survival rates than WT after 3 and 7 days of CDT (Fig. 2b). These data support the hypothesis that dormancy status is negatively correlated with the longevity of mature dry seeds.

The effect of priming on germination speed and longevity was also determined using the same mutants (Fig. 2a). The priming treatment itself increased germination speed for all genotypes. In addition, germination rates became synchronous for all the genotypes suggesting that the priming treatment reduced ABA-mediated seed dormancy. When the primed seeds were subjected to CDT, the survival rates of all genotypes were similarly reduced depending on the duration subjected to CDT and no significant differences were observed between them (Fig. 2c). These results indicate that the dormancy status of seeds prior to priming does not affect the reduction of longevity resulting from priming.

### Natural variation in seed longevity after priming

**Figure 3:**
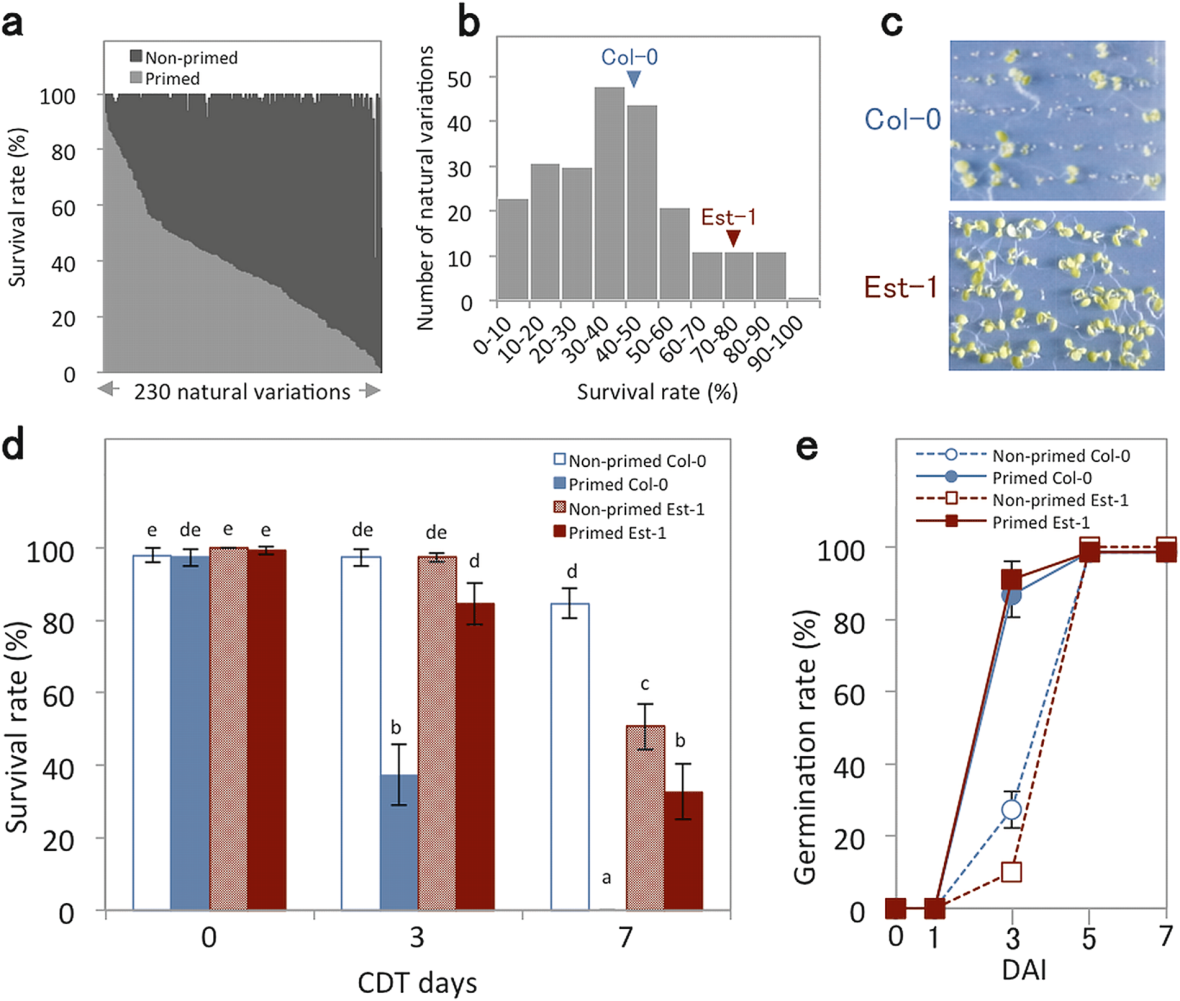
Seed longevity of *Arabidopsis* natural variants. (**a**) Survival rates of 230 *Arabidopsis* natural variations subjected to CDT for 3 days before (non-primed; dark grey bars) and after (primed; light grey bars) priming. (**b**) Frequency distribution of survival rates among the natural variants. (**c**) Photographs of Col-0 and Est-1 seedlings developed after priming and subsequent CDT for 3 days. (**d**) Survival rates of Col-0 and Est-1 before (non-primed) and after (primed) priming and subsequent CDT for 0, 3 and 7 days. (**e**) Germination speed of Col-0 and Est-1 before (non-primed) and after (primed) priming without CDT determined as the number of seedlings with green cotyledons. For (**d**) and (**e**), values presented are means ± SD of three replicates. Different letters in (**d**) indicate significant differences (P < 0.05, Tukey-Kramer tests).

To understand the genetic basis underlying the effect of priming on seed longevity, survival rates of primed and non-primed seeds were compared after CDT for 230 *Arabidopsis* natural variants (Fig. 3a and Supplementary Table S1). Among the accessions tested, we focused on Est-1 (Rd-0)^17^ that exhibits a relatively higher resistance to CDT than the reference accession Col-0 after priming (Fig. 3b and c). Survival rates of non-primed Est-1 and Col-0 seeds after 3 days of CDT were comparable, however, non-primed Est-1 seeds exhibited lower survival rates than Col-0 seeds when compared at 7 days of CDT (Fig. 3d). After priming, survival rates of Col-0 seeds were reduced by more than 60% on 3 days of CDT whereas those of Est-1 seeds were reduced by less than 20%. At 7 days of CDT, none of the primed Col-0 seeds were able to establish healthy seedlings in contrast to 33% of Est-1 seeds. These data suggest that Col-0 and Est-1 differ in mechanisms occurring during priming that subsequently impact seed longevity. In non-CDT aged seeds, the germination speed of both accessions either with or without priming was equivalent (Fig. 3e), which indicates that the dormancy status and germination speed of Col-0 and Est-1 were comparable.

### QTL analysis of seed longevity after priming

**Figure 4:**
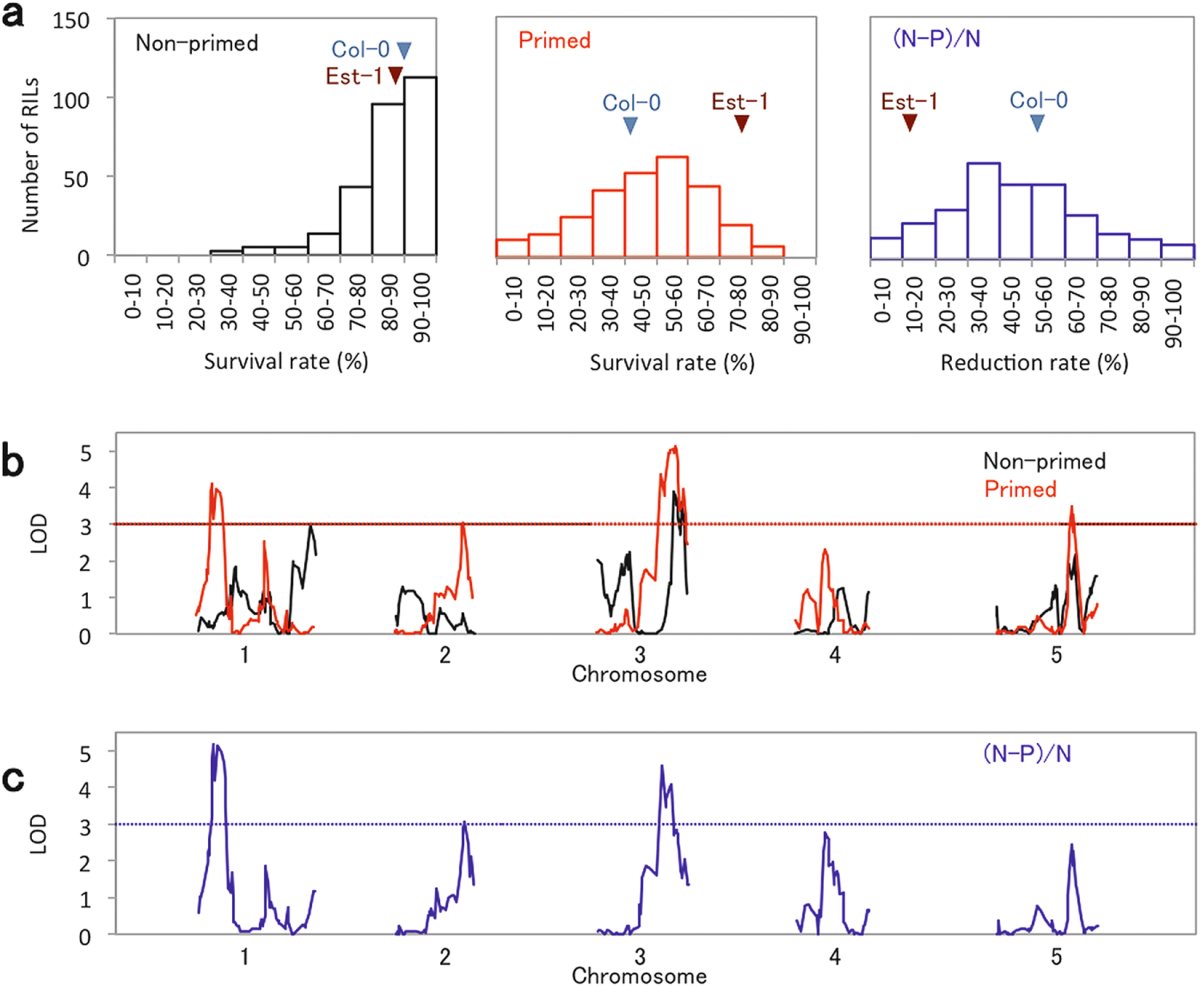
Identification of QTLs for seed longevity. (**a**) Frequency distributions of survival rates in 279 RILs before (non-primed; left panel) and after (primed; middle panel) priming after 3 days of CDT. Reduction in survival rates caused by priming are also shown [(N − P)/N; right panel]. (**b**) QTL mapping of seed longevity determined as survival rates after 3 days of CDT before (non-primed) and after (primed) priming. (**c**) QTL mapping of seed longevity determined as reduction of survival rates by priming [(N − P)/N]. Dotted lines in (**b**) and (**c**) indicate threshold (LOD = 3, P < 0.026 from 10,000-time permutation).

To identify loci that account for the variations in seed longevity between the two accessions, QTL analysis was performed using 279 RILs derived from a cross between Col-0 and Est-1, which had been genotyped for 224 molecular markers^18^. Survival rates for seeds of the RILs following a CDT of 3 days were determined before and after priming (Fig. 4a and Supplementary Table S2). No clear correlation was apparent between seed longevity before and after priming (Supplementary Fig. S2). Based on the survival rate phenotypes, QTLs associated with seed longevity before and after priming were mapped using the interval mapping method (Fig. 4b). Comparison of significant QTLs indicated an overlap of a major QTL detected on chromosome (chr) 3 for non-primed and primed seeds. Other major QTLs were also identified on the lower arm of chr 1 for non-primed seeds and for primed seeds, on the upper arm of chr 1, lower arm of chr 2 and lower arm of chr 5. These suggest that seed longevity of non-primed and primed seeds were determined largely by different genetic factors although longevity at the initial state in mature dry seeds influences seed longevity after priming to some extent. To eliminate the effects of primary longevity in non-primed seeds, we calculated the reduction rates of seed longevity caused by priming in the RILs [(Non-primed) − (primed)/(Non-primed) × 100] (Supplementary Table S2), and mapped QTLs based on this parameter (Fig. 4c). This validated three major QTLs associated with the reduction of seed longevity by priming on chr 1 (LOD 5.17), chr 2 (LOD 3.05) and chr 3 (LOD 4.60). For all these QTLs, Col-0 alleles contributed to the loss of seed longevity in response to priming (Supplementary Fig. S3). To evaluate the explained variance (%), we also analyzed these QTLs with the multiple QTL model and the results are summarized in Supplementary Table S3. The intervals covered by the three QTLs contained a large number of annotated genes (Supplementary Table S4). Identification of causal genes for QTLs by fine mapping generally requires a relatively long time. Thus, in the present study, we rather focused on the characterization of molecular mechanisms involved in the loss of seed longevity during priming than the identification of the causal genes.

### Detection of priming responsive genes by RNA-Seq

**Figure 5:**
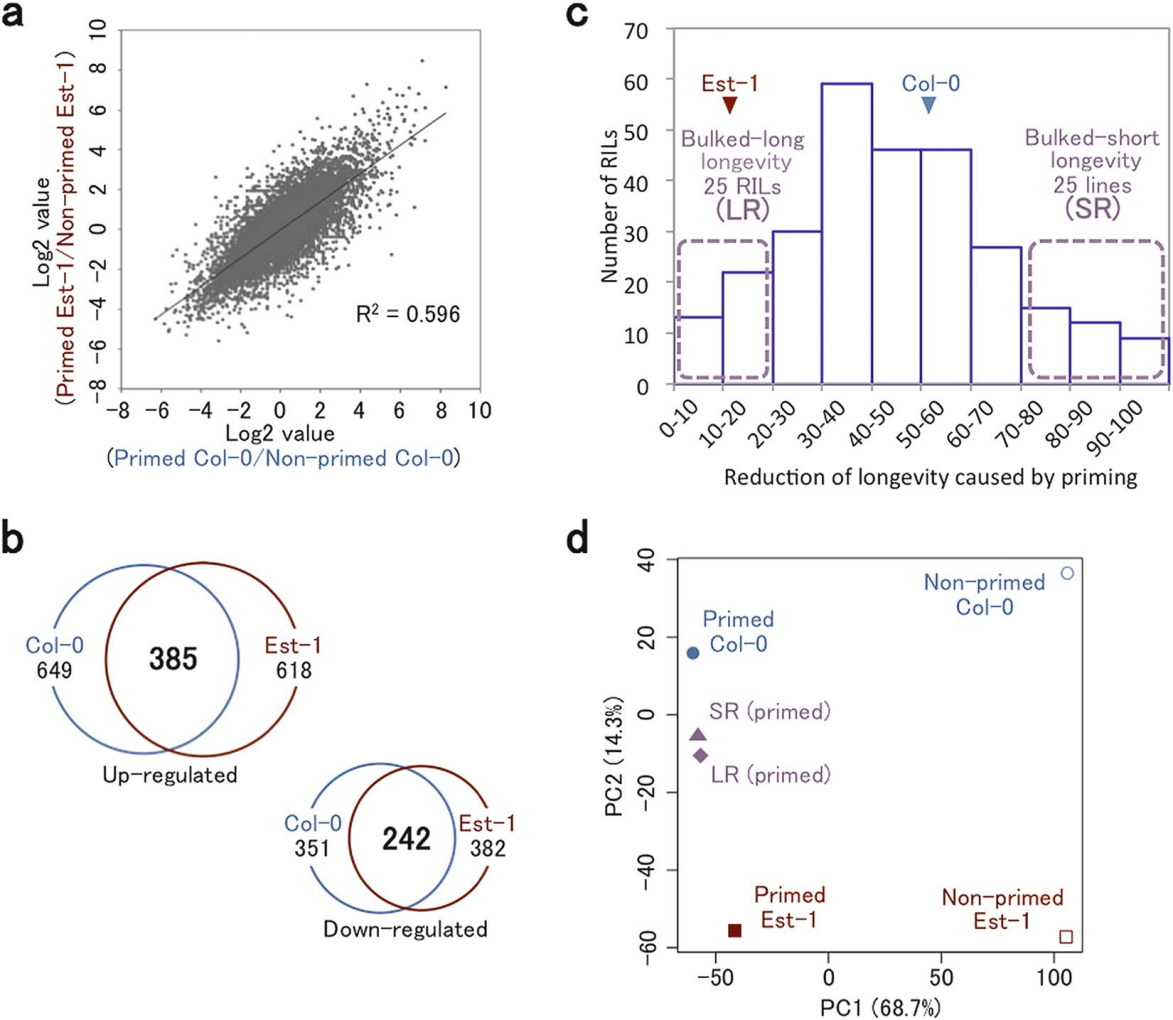
RNA-Seq analysis with seeds having different longevity. (**a**) Effect of priming on transcriptomes of Col-0 and Est-1 seeds. Fold changes in the gene expression levels by priming were compared between Col-0 and Est-1. (**b**) Comparison of top 1,000 genes whose expression was up- or down-regulated by priming between Col-0 and Est-1. (**c**) Frequency distribution of reduction rates of seed longevity after priming for the population of 279 RILs. The top 25 RILs with the greatest reduction were bulked as short RILs (SR). The top 25 RILs with the smallest reduction were bulked as long RILs (LR). (**d**) PCA of six transcriptome data obtained by RNA-Seq.

To better understand the molecular events associated with priming, transcriptome analysis was performed by RNA-Seq using non-primed and primed seeds of Col-0 and Est-1. A positive correlation (R^2^ = 0.596) was found between the effects of priming on gene expression between Col-0 and Est-1 (Fig. 5a), indicating that many genes were commonly up- or down-regulated in both accessions during priming. These are tentatively named ‘priming responsive genes’ here and were determined using the list of top 1,000 genes whose expression was changed during priming in Col-0 and Est-1 and the TCC package^19^. From these, 385 genes were commonly up-regulated in Col-0 and Est-1 and termed ‘priming up-regulated genes’ (Fig. 5b and Supplementary Table S5), while 242 genes were down-regulated in both accessions and termed ‘priming down-regulated genes’ (Fig. 5b and Supplementary Table S6). To obtain an overview of the physiological processes in which the priming responsive genes are involved, Gene Ontology (GO) enrichment analysis for biological processes was performed using the PANTHER Classification System^20^. No significant enrichment of GO terms was observed for priming down-regulated genes, while 17 GO terms including ‘response to oxygen-containing compound’, ‘response to toxic substance’, ‘translation’, ‘response to acid chemical’ and ‘response to karrikin’ were significantly enriched in priming up-regulated genes (Supplementary Table S7).

### Bulked transcriptome analysis using RILs

In addition to the priming responsive genes that were similarly regulated in Col-0 and Est-1, there were many genes whose expression levels differed between the two accessions. The latter might contain key regulatory genes that contribute to the difference in seed longevity between Col-0 and Est-1 under the control of detected QTLs. To efficiently extract possible candidates for these key regulatory genes, we employed ‘bulked transcriptome analysis’^21, 22^. This approach normalizes the variability among RILs that is not related to longevity by analysis of RNA from bulks of RILs with similar phenotypes. Based on the survival rate phenotypes after priming (Fig. 4a), 25 RILs that were relatively tolerant and 25 that were relatively sensitive to CDT were grouped as bulked-long RILs (LR) and bulked-short RILs (SR), respectively (Fig. 5c), and RNA-Seq were performed on bulks of the primed seeds. Principal component analysis (PCA) was then performed with the 6 transcriptome datasets (non-primed Col-0, non-primed Est-1, primed Col-0, primed Est-1, primed LR and primed SR) (Fig. 5d). The first dimension (PC1) represented variation due to the effects of priming whereas the second dimension (PC2) reflected differences between Col-0 and Est-1, being consistent with the observation that many genes were commonly up- or down- regulated during priming in both Col-0 and Est-1 (Fig. 5a). As expected, only minor differences were observed between the transcriptomes of LR and SR after priming compared to the differences between primed Col-0 and Est-1. In addition, the transcriptomes of LR and SR after priming were intermediate between primed Col-0 and Est-1. Comparison of primed seeds with longer (Est-1 and LR) or shorter longevity (Col-0 and SR) identified 46 genes that are differentially expressed according to following criteria: (i) in the top 100 genes differentially expressed between LR and SR, (ii) more than 1.5-fold differences in normalized read counts between LR and SR, and (iii) more than 1.2-fold differences in normalized read counts between Col-0 and Est-1. Of these, 11 genes were highly expressed in the long life populations (Table 1) and 35 genes highly expressed in the short life populations (Table 2). Comparison of the 46 genes and three major QTL regions for the loss of seed longevity during priming (Fig. 4c) revealed that five (AT1G12650, AT1G12805, AT1G16550, AT1G19540, and AT1G20390), six (AT2G44460, AT2G45400, AT2G45510, AT2G45560, AT2G46450 and AT2G46950) and five (AT3G46000, AT3G46610, AT3G47350, AT3G48580 and AT3G49570) genes were located within the QTL regions on chr 1, 2 and 3, respectively (Tables 1 and 2). In addition, three genes related to brassinosteroid (BR) synthesis or signaling [*BEN1* (AT2G45400)^23^, *DWF1* (AT3G19820)^24^ and *EXO* (AT4G08950)^25^] and four cell wall modification-related genes [*TRG1*/*XYL1* (AT1G68560)^26, 27^, *EXPA1* (AT1G69530)^28^, *EXPA2* (AT5G05290)^29^ and *DUF642* (AT5G11420)^30^] were more highly expressed in the short life populations compared to the long life populations (Table 2). This implies that BR and cell wall-related genes could participate in the regulatory networks determining longevity of primed seeds possibly under the control of the causal genes responsible for the QTLs.

**Table 1:** Genes highly expressed in primed seeds of high longevity genotypes (bulked-long RILs and Est-1).

Gene ID	Gene expression levels (Normalized read counts)				Annotation	Overlapped QTL
	SR^a^	LR^b^	Col-0	Est-1		
AT1G12130	73.3	158.4	41.3	218.6	Flavin-binding monooxygenase family protein	—^c^
AT1G16550	42.2	93.5	40.3	79.3	Pseudogene, putative cell division cycle protein	chr 1
AT1G64900	582.1	880.0	218.4	1060.7	CYP89A2	—
AT2G44460	23.8	66.0	10.3	16.1	BGLU28 (beta glucosidase28)	chr 2
AT2G46950	187.0	354.2	21.6	500.4	CYP709B2	chr 2
AT3G44215	145.8	430.1	0.9	606.4	Copia-like retrotransposon family protein	—
AT3G48580	131.1	253.0	100.3	422.1	XTH11 (xyloglucan endotransglucosylase/hydrolase11)	chr 3
AT3G49570	229.2	387.2	159.4	350.4	LSU3 (response to low sulfur3)	chr 3
AT3G60140	282.3	575.3	127.5	311.8	BGLU30 (beta glucosidase30)	—
AT4G13180	463.8	706.2	476.3	595.7	NAD(P)-binding Rossman-fold superfamily protein	—
AT5G48850	132.0	218.9	46.9	63.2	SDI1 (sulfur deficiency-induced1)	—

^a^SR; bulked-short RILs. ^b^LR; bulked-long RILs. ^c^No overlap with the QTLs.

**Table 2:** Genes highly expressed in primed seeds of short longevity genotypes (bulked-short RILs and Col-0).

Gene ID	Gene expression level (Normalized read counts)				Annotation	Overlapped QTL
	SR^a^	LR^b^	Col-0	Est-1		
AT1G01300	265.8	172.7	300.9	162.9	Eukaryotic aspartyl protease family protein	—^c^
AT1G03106	797.5	513.7	927.2	455.4	Unknown protein	—
AT1G12650	44.0	14.3	55.3	4.3	Unknown protein	chr 1
AT1G12805	240.2	101.2	496.9	77.1	Nucleotide binding protein	chr 1
AT1G19540	235.6	93.5	309.4	76.1	NmrA-like negative transcriptional regulator family protein	chr 1
AT1G20390	62.3	9.9	73.1	0.0	Gypsy-like retrotransposon	chr 1
AT1G22110	138.4	73.7	302.8	114.6	Structural constituent of ribosome	—
AT1G27340	99.0	46.2	74.1	61.1	LCR (leaf curing responsiveness)	—
AT1G68560	268.6	141.9	237.2	49.3	TRG1 (thermoinhibition resistant1)	—
AT1G69530	766.3	454.3	1154.1	173.6	EXPA1 (expansin A1)	—
AT1G80530	238.3	149.6	229.7	186.4	Major facilitator superfamily protein	—
AT2G10410	286.9	156.2	410.6	0.0	SADHU1-1 (sadhu non-coding retrotransposon1-1)	—
AT2G14878	610.5	212.3	1054.7	9.6	Non-coding RNA	—
AT2G14960	61.4	26.4	37.5	13.9	GH3.1 (Protein similar to IAA-amido synthases)	—
AT2G16880	76.1	36.3	58.1	46.1	Pentatricopeptide repeat superfamily protein	—
AT2G20670	33.0	8.8	17.8	8.6	DUF506 (Protein of unknown function)	—
AT2G45400	18.3	2.2	15.9	4.3	BEN1 (BRI1-5 ENHANCED1)	chr 2
AT2G45510	256.7	166.1	239.1	159.6	CYP704A2	chr 2
AT2G45560	246.6	134.2	298.1	48.2	CYP76C1	chr 2
AT2G46450	55.0	22.0	101.3	2.1	CNGC12 (cyclic nucleotide-gated channel12)	chr 2
AT3G09210	83.4	38.5	56.3	34.3	PTAC13 (plastid transcriptionally active13)	—
AT3G19820	74.3	27.5	58.1	48.2	DWF1 (DWARF1)	—
AT3G27770	129.3	67.1	120.9	51.4	Unknown protein	—
AT3G42052	19.3	2.2	16.9	0.0	Gypsy-like retrotransposon	—
AT3G43580	63.3	20.9	45.9	0.0	Beta-galactosidase related protein	—
AT3G43955	61.4	20.9	53.4	0.0	Copia-like retrotransposon	—
AT3G44430	136.6	36.3	165.0	0.0	Unknown protein	—
AT3G46000	221.8	138.6	190.3	122.1	ADF2 (actin depolymerizing factor2)	chr 3
AT3G46610	80.7	36.3	92.8	16.1	Pentatricopeptide repeat superfamily protein	chr 3
AT3G47350	82.5	27.5	85.3	0.0	HSD2 (hydroxysteroid dehydrogenase2)	chr 3
AT4G08950	193.4	105.6	41.3	30.0	EXO (exordium)	—
AT4G10390	30.3	7.7	21.6	10.7	Protein kinase superfamily protein	—
AT4G36880	1181.6	716.1	1543.1	313.9	CP1 (cysteine proteinase1)	—
AT5G05290	102.7	35.2	134.1	8.6	EXPA2 (expansin A2)	—
AT5G11420	345.6	192.5	354.4	75.0	DUF642 (protein of unknown function)	—

^a^SR; bulked-short RILs. ^b^LR; bulked-long RILs. ^c^No overlap with the QTLs.

### Effects of BR on seed longevity after priming

**Figure 6:**
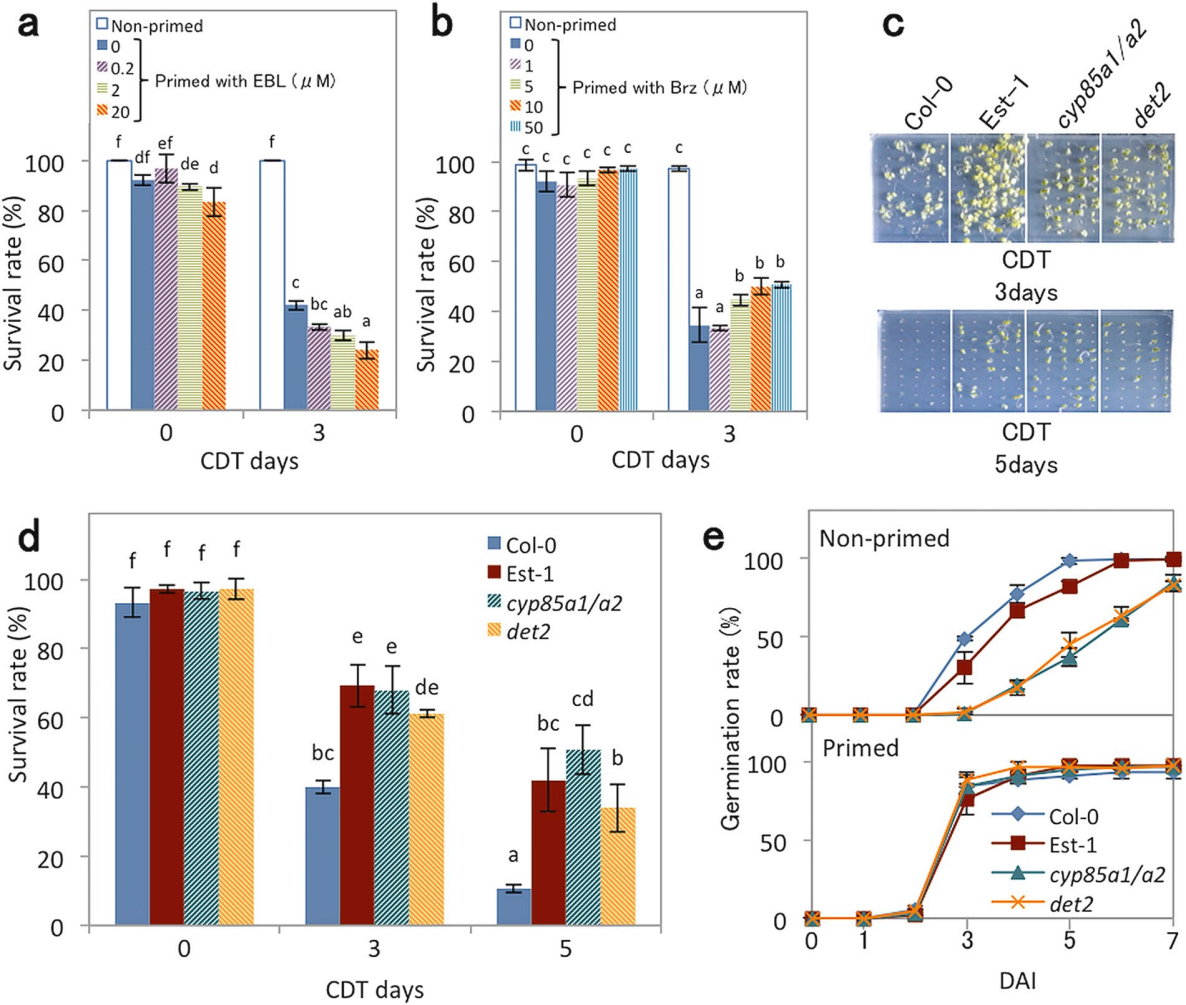
Effect of BR on seed longevity. (**a**) Survival rates of wild type Col-0 non-primed seeds or seeds primed with 0, 0.2, 2 or 20 μM EBL after 0 or 3 days of CDT. (**b**) Survival rates of wild type Col-0 non-primed seeds or seeds primed with 0, 1, 5, 10 or 50 μM Brz after 0 or 3 days of CDT. (**c**) Germination of wild type Col-0, Est-1, *cyp85a1 cyp85a2* (*cyp85a1*/*a2*) and *det2* after priming and subsequent CDT for 3 or 5 days. Photos were taken after 7 days of imbibition. (**d**) Survival rates of wild type Col-0, Est-1, *cyp85a1*/*a2* and *det2* after priming and subsequent CDT for 0, 3 or 5 days. (**e**) Germination speed of wild type Col-0, Est-1, *cyp85a1*/*a2* and *det2* before (non-primed; upper panel) and after (primed; lower panel) without CDT. For (**a**), (**b**), (**d**) and (**e**), values presented are means ± SD of three replicates. Wild type Col-0 (Col-0). Different letters in (**a**), (**b**) and (**d**) indicate significant differences (P < 0.05, Tukey-Kramer tests).

To examine the effect of BR on longevity of primed seeds, Col-0 seeds were subjected to CDT after priming in the presence of a bioactive BR (24-epibrassinolide; EBL) or a BR biosynthesis inhibitor (brassinazole; Brz^31^) (Fig. 6a and b). EBL promoted the reduction of longevity after priming in a concentration dependent manner (Fig. 6a). Conversely, when Brz was present at concentrations higher than 5 μM during priming, seed longevity was reduced less (Fig. 6b). Furthermore, the reduction of endogenous levels of BR in the BR-deficient mutants *cyp85a1 cyp85a2* (*cyp85a1/a2*)^32^ and *det2* ^33^, in the Columbia, also increased CDT survival rates after priming to levels similar to Est-1 (Fig. 6c and d), although *cyp85a1/a2* was significantly more resistant to CDT than other genotypes even before priming (Supplementary Fig. S4). These results suggest that BR promotes the loss of seed longevity during priming. The germination speed of *cyp85a1/a2* and *det2* before priming was slower than that of Col-0 and Est-1, whereas priming treatment efficiently enhanced the speed of both *cyp85a1/a2* and *det2*, to levels similar to Col-0 and Est-1 (Fig. 6e).

### Influence of the seed coat on longevity of primed seed

**Figure 7:**
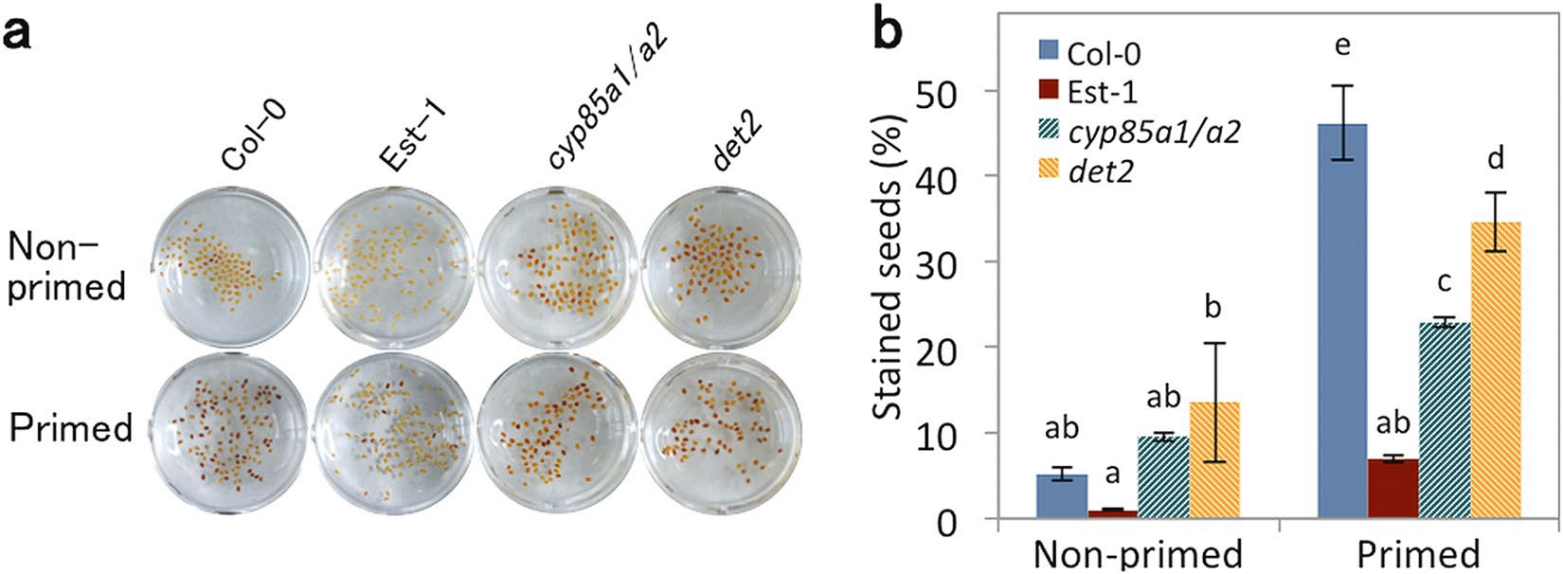
Changes in seed coat permeability after priming. (**a**) Tetrazolium staining of wild type Col-0 (Col-0), Est-1, *cyp85a1 cyp85a2* (*cyp85a1*/*a2*) and *det2* seeds before (non-primed) and after (primed) priming. (**b**) Numbers of stained seeds (red colored seeds) before (non-primed) and after (primed) priming for Col-0, Est-1, *cyp85a1*/*a2* and *det2*. Values presented are means ± SD of three replicates. Different letters indicate significant differences (P < 0.05, Tukey-Kramer tests).

Seed longevity largely relies on viability of the embryos. Nevertheless, although the seed coat that surrounds embryos is formed of maternally derived dead cell layers, mutants that have defects in seed coat properties (e.g. structure and pigmentation) exhibited reduced seed longevity^6, 34^. This led us to investigate the contribution of the seed coat to the loss of seed longevity during priming. Since it has been reported that seed coat permeability can be evaluated by tetrazolium staining^34^, we incubated primed and non-primed seeds of Col-0, Est-1, *cyp85a1/a2* and *det2*, with tetrazolium salts for 24 h (Fig. 7a). Non-primed seeds of all genotypes were almost not stained and did not show notable differences in the number of stained seeds (Fig. 7b). In contrast, after priming, significantly more seeds were stained in Col-0, but not in Est-1, suggesting that changes in seed coat properties might be associated with the reduction of seed longevity during priming. In *cyp85a1/a2* and *det2* the numbers of stained seeds were also increased by priming, however, they were significantly less than for wild type Col-0, implying that seed coat permeability might be at least partially regulated by BR during priming.

## Discussion

Seed priming is a treatment used to enhance seed performance prior to their commercialization. Although the treatment often affects post germination events such as seedling growth (vigor) and resistance to biotic and abiotic stress, here we have focused on the relationship between the promotion of germination and the loss of longevity caused by the treatment. Germination can be considered as the process during which a mature dry seed gradually loose its seed-specific characters and become a seedling, and the loss of seed longevity is a part of the program. Thus, studying the molecular mechanisms involved in the loss of seed longevity during priming is important not only for agricultural applications but also for understanding basic seed physiology. In the present study, hydropriming was used in combination with stratification (or thermoprming) for the assay because it is simple and sufficient to determine the effect of priming on seed germination and longevity (Fig. 1).

QTL analysis using six RIL populations has previously been used to demonstrate that seed dormancy and longevity in mature dry seeds is negatively correlated with DOG QTLs^13^. Similarly, our present study showed that the dormancy status of Col-0, *aba2* and c*yp707a2* seeds before priming was negatively correlated with seed longevity, although this is contradictory to the observation that the ABA deficient *aba1-5* mutant had shorter longevity than the wild type Columbia background under natural aging. No significant differences were, however, observed in longevity when seeds were subjected to priming, indicating that the reduced survival rate after priming was independent of initial seed dormancy levels.

In this study, we identified an accession, Est-1, which shows longer longevity after priming than the reference accession Col-0 (Fig. 3d). Interestingly, longevity of Est-1 seeds before and after priming did not change drastically, compared to Col-0 seeds, which rapidly lost their longevity upon priming. This suggests that Col-0 and Est-1 have different germination programs. QTL mapping with RILs derived from a cross between Col-0 and Est-1 identified three genomic regions associated with the reduction of longevity during priming. Even though the two accessions showed large differences in this trait, it is generally difficult to extract important gene expression profiles responsible for a unique character by just simply comparing transcriptomes of different accessions. Transcriptomes of germinating seeds are complex, because proteins required for germination are translated not only from mRNAs newly transcribed upon imbibition, but also from mRNA templates that are synthesized during seed maturation and pre-existing in the mature dry seeds (stored mRNA or long-lived mRNA^35–37^). In addition, transcriptomes in dry seeds of less-dormant Columbia and dormant Cvi accessions resemble each other, suggesting that patterns of stored mRNA accumulation reflect neither the degree of dormancy nor germination potential, but rather the developmental context such as seed maturation^38^. Here, a bulked transcriptome analysis using RILs, allowed the identification of BR and cell wall-related genes that may contribute to the difference in seed longevity between the two accessions. In effect, BR-deficient mutants exhibited improved longevity after priming compared to WT and priming with a bioactive BR (EBL) and a BR biosynthesis inhibitor (Brz) reduced or prolonged seed longevity, respectively. These data strongly suggest that BR is involved in the loss of longevity during priming and that bulked transcriptome analysis with RILs can be an effective approach for predicting genes involved in traits differing between accessions. Several genes associated with short longevity after priming were found in the QTL regions associated with the loss of seed longevity by priming (Tables 1 and 2). However, a number of single nucleotide polymorphisms (SNPs) were present in the ORF plus 1 kb upstream regions of these genes (Supplementary Table S8). Further fine mapping will be required to identify the causal genes and SNPs responsible for the genetic variations. Characterization of mutants defective in the candidate gens will be also required to determine their involvement in the loss of seed longevity during priming. These studies would contribute to improve seed longevity after priming in crop species.

BR is a plant hormone that regulates a wide range of developmental and physiological processes^39, 40^. Although little is known about the relationship between BR and seed longevity, it has been shown that BR regulates seed dormancy and/or germination. In *Arabidopsis*, germination of the BR deficient mutant *det2* and the BR-insensitive mutant *bri1* is more strongly inhibited by ABA than WT^41^. The same study also showed that BR treatment induces germination of the severe GA-deficient mutant *ga1-3*, which normally requires exogenous GA for germination. These data suggest that BR promotes seed germination and are consistence with our observation that BR deficient mutants *cyp85a1/a2* and *det2* exhibited reduced germination rates compared to WT in the absence of priming (Fig. 6e). We speculate that BR mediates part of the germination process induced during priming, as a result this is only partially initiated in BR-deficient mutants so that seed longevity after priming is less affected compared to WT. Since the effect of BR on seed germination is opposite to that of ABA, one might speculate that BR indirectly affects seed longevity during priming by mediating ABA signaling. However Fig. 2c shows that dormancy levels (or germination speed) determined by ABA do not affect the reduction of longevity during priming. Thus it is likely that the effect BR on the reduction of seed longevity during priming is independent from its effects on ABA signaling.

Cell expansion is dependent on the tension of cell walls of which major components are the cellulose microfibrils and the pectin/hemicellulose matrix. Loosening of the wall allows water influx to drive cell expansion and generate cellular turgor pressure^42^. Embryonic cell expansion and endosperm weakening during seed germination are controlled by cell wall modification enzymes^26^. We found that four cell wall modification-related genes *TRG1/XYL1* (AT1G68560), *EXPA1* (AT1G69530), *EXPA2* (AT5G05290) and *DUF642* (AT5G11420) were highly expressed in seeds of bulked RILs that had relatively short longevity after priming (Table 2). *TRG1/XYL1* encodes an α-xylosidase that is required for xyloglucan maturation and is supposed to be a germination suppressor, because mutants defective in the corresponding gene showed reduced dormancy, thermoinhibition-resistant germination, and resistance to the GA biosynthesis inhibitor paclobutrazol during germination^26, 27^. The phenotypes of *trg1*/*xyl1* mutant are possibly associated with altered endosperm cell wall composition that impact on its resistance^26^. In contrast, overexpression of *DUF642* increased pectin methylesterase (PME) activity and promoted testa and endosperm rupture in response to matrix based priming^30^. It has been reported that DUF642 interacts with catalytic domain of AtPME3^43^. This is in accord with the promotion of testa rupture and enhanced testa permeability observed on exogenous treatment of garden cress (*Lepidium sativum*) seeds with PME during imbibition^44^. PME controls the methylesterification status of pectin and thereby determines the biophysical properties of cell walls. Interestingly, a relationship between PME action and BR signaling was previously reported. Several growth defects caused by the over-expression of a PME inhibitor, AtPMEI5, were suppressed by a mutation in the BR receptor BRI1^45^. BR has previously been implicated in the activation of a large number of genes encoding cell wall-related enzymes, such as cellulose synthase, pectinesterases, xyloglucosyl transferases, and expansins^39^. *EXP1* was up-regulated by BR^39^, while mutants defective in *EXPA2* showed delayed germination^29^. These observations suggest that BR-mediated cell wall modification influence the reduction of seed longevity during priming. While *TRG1/XYL1* is a negative regulator of germination, *DUF642* and *EXPA2* promote germination, suggesting that diverse cell wall modifications have different effects on germination and longevity.

Tetrazolium staining demonstrated that the priming treatment increased seed coat permeability (Fig. 7). This is in accord with the high seed coat permeability and reduced seed longevity exhibited by *transparent testa* (*tt*) mutants affected in the synthesis of the flavonoids anthocyanins and proanthocyanidins^34^. Moreover, *wrky3* and *nfxl1* mutants defective in defense-related transcription factors also have increased seed coat permeability and impaired acquisition of longevity during seed maturation^46^. These suggest a link between seed coat functions and longevity. The *Arabidopsis* TT10 laccase is present in the young colorless seed coat, and the oxidation of soluble proanthocyanidins into quinonic compounds by TT10 laccase might increase their capacity to bind to the cell wall during seed desiccation. It has also been pointed out that the formation of antimicrobial quinones and insoluble polymers results in the reinforcement of the seed coat as a barrier to water/oxygen permeation, mechanical damage and biotic/abiotic stresses^47^. In the present study, we showed that improved seed survival after priming is associated with a better maintenance of seed coat impermeability in Est-1, *cyp85a1*/*a1* and *det2* compared with WT Col-0. As mentioned above, BR-mediated cell wall modifications might be involved in the loss of seed longevity during priming. We propose that BR directly or indirectly modulates seed coat permeability during priming.

## Methods

### Plant materials and growth conditions

The *aba2* mutant (*aba2-2*) used in this study was isolated by Nambara *et al*. (1998)^14^. The *cyp707a1*, *cyp707a2* and *cyp707a3* mutants (*cyp707a1-1*, *cyp707a2-2* and *cyp707a3-1*, respectively) were isolated as described previously^15, 16^. The *cyp85a1/a2* mutant (*cyp85a1-1 cyp85a2-2*) was isolated by Nomura *et al*.^32^. To isolate the *det2* mutant used in this study, seeds for the T-DNA insertion line CS873725 were obtained from the *Arabidopsis* Biological Resource Center (ABRC) (http://abrc.osu.edu/). The homozygous mutant was selected from its visual phenotype. T-DNA insertion of the *det2* mutant was confirmed by PCR using primers BP (5′-TCAGAAATGGATAAATAGCCTTGCTTC-3′) and RP (5′-CTTGGCAATGTACCACTTGTGACTC-3′) based on their ability to amplify a fragment, and homozygous insertion was confirmed by PCR using primers LP (5′-CCAAGTGGCCAAAACCTAGCTTC-3′) and RP based on their inability to amplify a fragment. Col-0 was used as the wild type control for these mutants. The 230 *Arabidopsis* natural variations used in this study were obtained from the RIKEN BioResource Center (http://www.brc.riken.jp/). The set of 279 RILs derived from the Est-1 × Col-0 (CS39289) were obtained from *Arabidopsis* Biological Resource Center.

Seeds were surface-sterilized in a solution containing 5% (v/v) NaClO and 0.05% (v/v) Tween 20, rinsed with water, and sown on Murashige and Skoog media [one-half strength Murashige and Skoog salts, MES (0.5 g/l), pH 6.5] containing 0.8% (w/v) agar. After stratification at 4 °C for 3 days in the dark, plates were incubated at 22 °C under continuous light (approximately 10 W/m^2^). To propagate seeds, about 1-week-old seedlings were transplanted on pots containing vermiculite and Metro-mix 350 at a 3:1 ratio and grown in growth chambers at 22 °C under continuous white light and supplemented with nutrients (1/1,000 dilution of Hyponex; Hyponex Japan).

### Physiological analysis

For priming treatments, surface-sterilized seeds in 1.5 ml tubes were imbibed at 4 °C for 3 days in the dark and then at 22 °C for 12 h under the light. The seeds were dried on filter papers under a laminar flow cabinet for 12 h. For seed priming with EBL or Brz, the stock solutions (200 × concentration) prepared in DMSO were added to the water after sterilization, and water containing 0.5% (v/v) DMSO were used for control experiments.

To evaluate longevity, seeds were subjected to CDT by storing them in a sealed box with an open petri dish containing saturated KCl solution for 0 to 20 days at 37 °C. Relative humidity inside the box was maintained at 80%. Germination assays were performed on Murashige and Skoog media containing 0.8% (w/v) agar with 50 seeds in triplicates. Germination speed was defined as radicle emergence from seed coat or as opening of green cotyledons as described in the figure legends. Seed survival after CDT was defined as establishment of seedlings with green opened cotyledons at 7 DAI.

Seed coat permeability was assessed by staining 50 to 100 seeds with 1% (w/v) 2,3,5-triphenyltetrazolium chloride solution at 30 °C for 24 h. The number of red-colored seeds was counted under a stereomicroscope.

### QTL analysis

Survival rates of primed and non-primed seeds after 3 days of CDT were used as phenotype data of 279 RILs. Normalized seed longevity values after priming were calculated as follows: [(Non-primed seed longevity) − (Primed seed longevity)/(Non-primed seed longevity) × 100]. Genotype data of the RILs were obtained from The *Arabidopsis* Information Resource (ftp://ftp.arabidopsis.org/home/tair/Maps/). QTL analysis was performed using the R-qtl package^48^ implemented in R (http://www.r-project.org).

### Transcriptome analysis

Total RNA was prepared from 20 mg primed or non-primed seeds by acid phenol extraction and lithium chloride precipitation as previously described^49^. For bulked transcriptome analysis, the top 25 RILs with either longer or shorter longevity were selected for bulks. Total RNA was isolated from primed seeds of individual RILs, and 1 μg of the total RNA from 25 RILs was mixed as LR or SR samples, respectively. Preparation of mRNA-Seq library and sequencing were performed via a commercial service (Eurofins Genomics K.K., Tokyo, Japan). Paired-end libraries were sequenced (2 × 125 bp module) with Illumina Hiseq-2500 version 4 chemistry. Sequenced paired-end reads were mapped onto the *Arabidopsis* genome (TAIR version 10) obtained from Ensembl Plants (http://plants.ensembl.org/info/website/ftp/index.html) using the Bowtie2 (version 2.2.6)^50^. Reads mapped onto exon regions were counted using featureCounts (version 1.5.0-p1)^51^, and summarized in Supplementary Table S9. Normalization of read counts and ranking of differentially expressed genes were performed using the R package TCC^19^. Based on the normalized read counts, PCA was performed using the prcomp function of R. The GO enrichment analysis was performed using annotation data set ‘GO biological process complete’ (release 20161027) by the PANTHER Classification System (release 20160715)^20^ with the Bonferroni correction for multiple testing. SNPs information in Supplementary Table S8 was obtained from the 1001 Genomes portal (http://1001genomes.org/).

### Statistical analysis

Tukey-Kramer tests were used to determine significant differences in multiple comparisons of seed longevity and seed coat permeability.

### Data deposition

RNA-Seq data in this publication have been deposited in DRA (http://trace.ddbj.nig.ac.jp/dra) under accession numbers DRA005725.

### Data Availability

All data generated or analyzed during this study are included in this published article (and its Supplementary Information files) or are available from the corresponding author on reasonable request.

## Electronic supplementary material

Supplementary figures

Supplementary Tables
