# Supplementary material for: RNA-Seq using bulked recombinant inbred line populations uncovers the importance of brassinosteroid for seed longevity after priming treatments
Source: Sci Rep. 2017 Aug 14;7:8095. doi: 10.1038/s41598-017-08116-5 (PMC5556009; doi:10.1038/s41598-017-08116-5)
Supplement: Supplementary file 1 — Supplementary figures [file 41598_2017_8116_MOESM1_ESM.pdf]

RNA-Seq using bulked recombinant inbred line populations uncovers the importance of brassinosteroid for seed longevity after priming treatments

Naoto Sano[1], June-Sik Kim[2 #], Yoshihiko Onda[1], Takahito Nomura[3], Keiichi Mochida[1 4, 5], Masanori Okamoto[2 #], and Mitsunori Seo[1, *]

[1] RIKEN Center for Sustainable Resource Science, 1-7-22 Suehiro-cho, Tsurumi-ku, Yokohama, Kanagawa 230-0045, Japan.

[2] Arid Land Research Center, Tottori University, 1390 Hamasaka, Tottori 680-0001, Japan

[3] Center for Bioscience Research and Education, Utsunomiya University, 350, Mine-machi, Utsunomiya 321-8505, Japan

[4] Kihara Institute for Biological Research, Yokohama City University, 641-12 Maioka-cho, Totsuka-ku, Yokohama, Kanagawa 244-0813, Japan

[5] Institute of Plant Science and Resources, Okayama University, 2-20-1 Chuo, Kurashiki, Okayama 710-0046, Japan

[#] Present addresses: RIKEN Center for Sustainable Resource Science, 3-1-1 Koyadai, Tsukuba-shi, Ibaraki 305-0074, Japan (JS. K.); Center for Bioscience Research and Education, Utsunomiya University, 350, Mine-machi, Utsunomiya 321-8505, Japan (M.O.)

* Corresponding author: Mitsunori Seo, Email: mitsunori.seo@riken.jp

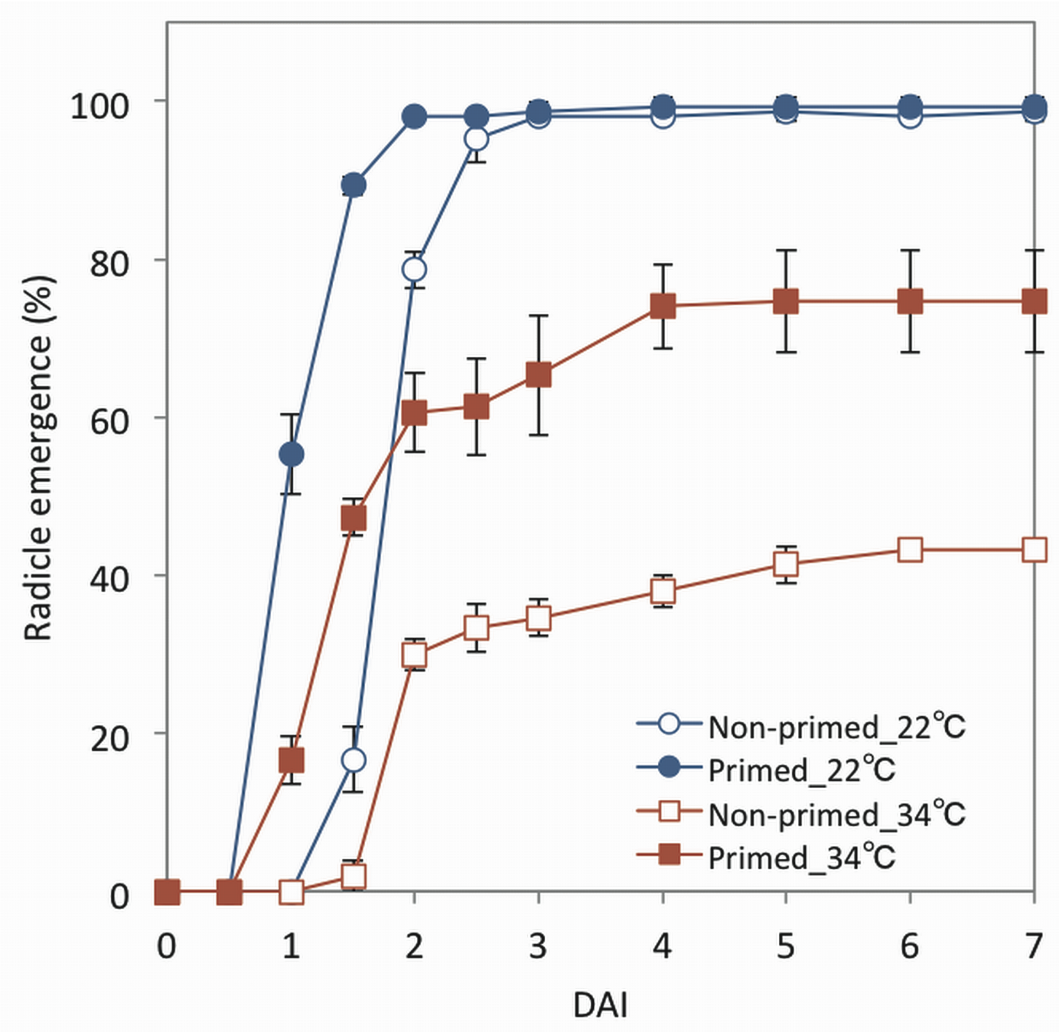

Supplementary Figure S1. Effect of priming on radicle protrusion at 34°C. Primed and non-primed Col-0 seeds were imbibed at 22°C or 34°C without CDT and the rates of radicle emergence from the seed coat were scored. Values presented are means ±SD of three replicates.

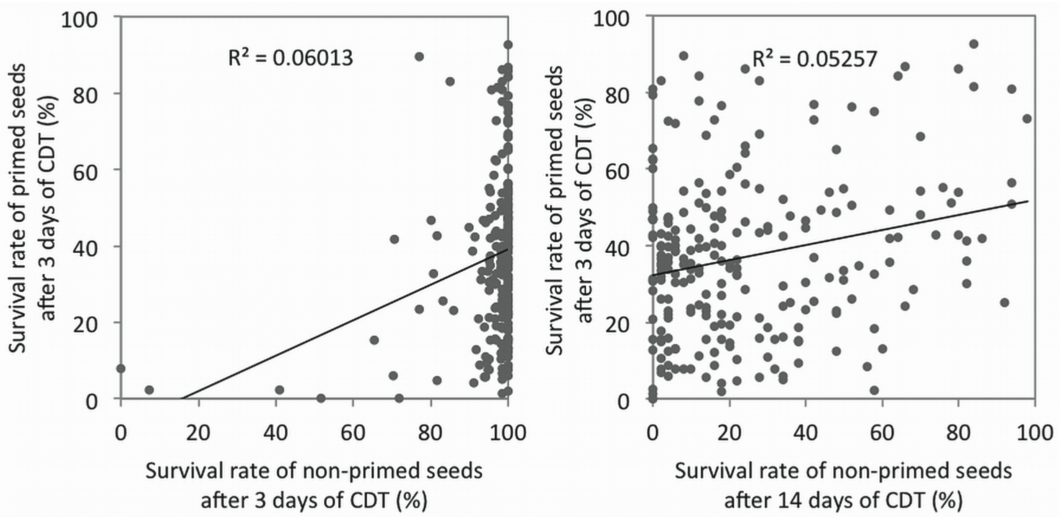

Supplementary Figure S2. Relationship between longevity of non-primed and primed seeds. Survival rates of non-primed and primed seeds after CDT were compared for 230 natural variations.

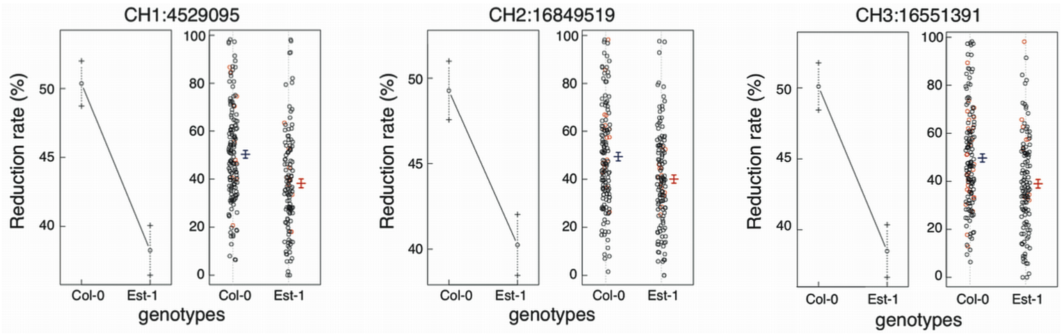

Supplementary Figure S3. Genetic effect of detected QTL markers on reduction rates of seed longevity after priming. The left panel presents phenotype means for each group defined by genotypes at each QTL markers. The error bars are plotted at +/- 1 standard error (SE). The right panel plots whole phenotypes for each group. Black and red circles refer the observed phenotypes and the imputed missing phenotypes, respectively. The blue and red indicators designate the same values to the left panel.

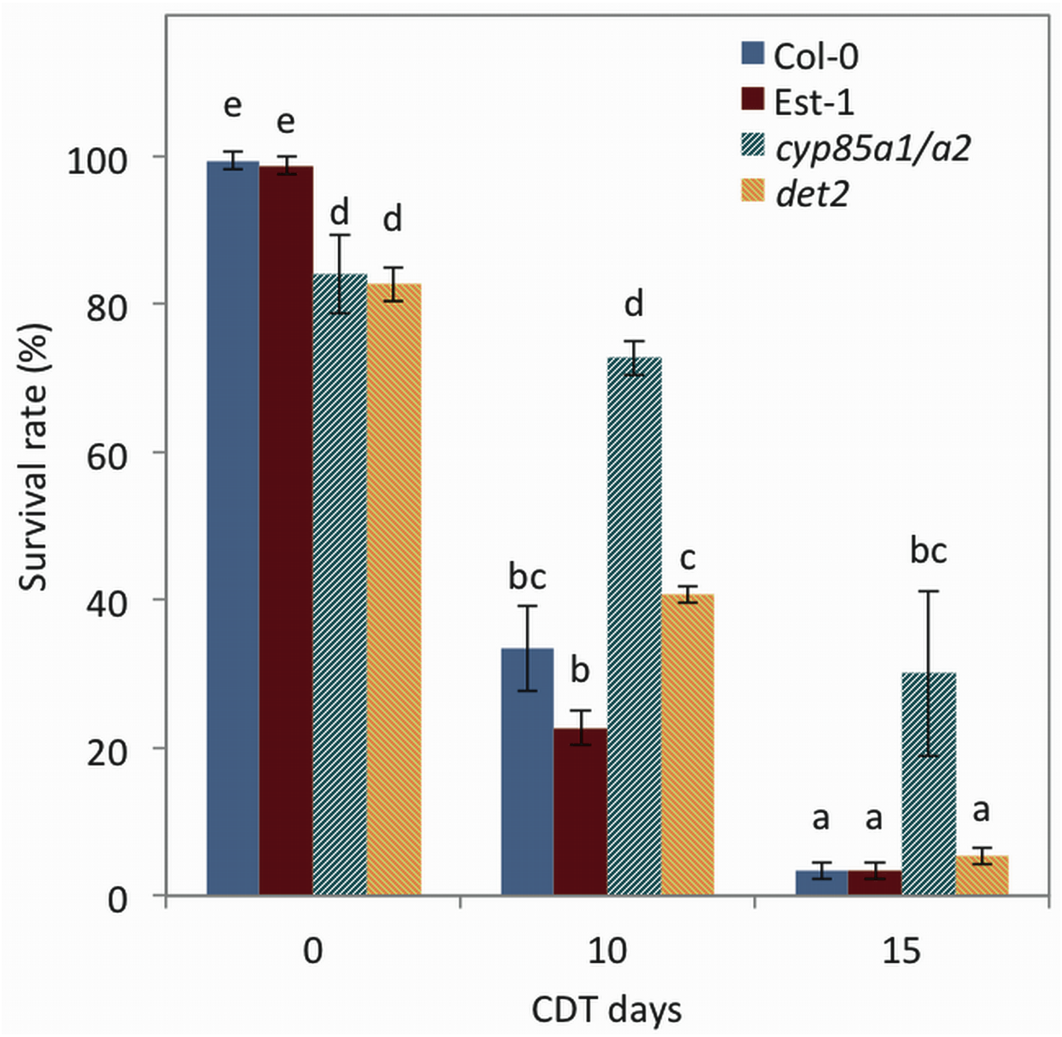

Supplementary Figure S4. Longevity of wild type Col-0, Est-1 *cyp85a1/a2* and *det2* seeds before priming. Survival rates of non-primed wild type Col-0 (Col-0), Est-1 *cyp85a cyp85a2* (*cyp85a1/a2*) and *det2* seeds after 0, 10, or 15 days of CDT are means ±SD of three replicates. Different letters indicate significant differences ($P < 0.05$, Tukey-Kramer tests).
